# Demographic, clinical and treatment characteristics of the carra registry systemic JIA cohort

**DOI:** 10.1186/1546-0096-12-S1-P64

**Published:** 2014-09-17

**Authors:** Ginger Janow, Laura Schanberg, Soko Setoguchi, Elizabeth D Mellins, Rayfel Schneider, Yukiko Kimura

**Affiliations:** 1 Pediatrics, Joseph M Sanzari Children's Hospital, Hackensack, USA; 2 Pediatrics, Duke University, Durham, USA; 3 Pharmacoepidemiology, Duke Clinical Research Institute, Durham, USA; 4 Pediatrics, Stanford University, Stanford, USA; 5 Pediatrics, Hospital for Sick Children, Toronto, Canada

## Introduction

Systemic Juvenile Idiopathic Arthritis (sJIA) is a disease with systemic inflammatory features as well as arthritis. Before biologic agents were available, a significant proportion of affected children suffered from poor outcomes. The Childhood Arthritis and Rheumatology Research Alliance (CARRA) Registry is a rich resource of information about pediatric rheumatic diseases including sJIA.

## Objectives

We aimed to: (1) describe the characteristics of sJIA children in the CARRA Registry; (2) identify medication usage trends; and (3) identify subgroups at increased risk for poor outcomes.

## Methods

54 active study sites in the US and Canada contributed 528 sJIA patients from 2010-2013. Patients were enrolled as a cross-sectional convenience sample, not an inception cohort. Only children with a complete set of core data were included in these analyses. We tested for subgroup differences among binary and continuous variables across the groups using a chi-square test or one-way analysis of variance (ANOVA) with the null hypothesis of multiple groups being equal.

## Results

435 had a complete data set at enrollment (median age 11yrs; age of symptom onset 4.6 yrs; first visit to pediatric rheumatologist 5.1 yrs). On the whole, disease activity was low: 14.9% rash, 6.7% fever, median joint count 0, and median physician global assessment 1. Significant changes in medication usage occurred over the study period 2010-2013: DMARD and TNF inhibitor use decreased, while IL-6 inhibitor use increased. There were significant differences among racial groups with African Americans (AA) having higher mean CHAQ scores, more poor/very poor quality of life scores and poorer ACR functional class (p 0.0004), despite no differences in medication use. Current and prior biologic use was more frequent in those with longer disease duration. Children diagnosed at a younger age (< 2 years old) had more frequent biologic use and lower overall well being scores. Joint damage on imaging was reported more often in those with younger age at diagnosis (p 0.0003). 308 had at least one follow up visit, 259 of whom had visits occurring at least 3 mos from enrollment. Trends towards improvement in disease activity measures were found in these 259 patients. Of 234 children with no systemic features at baseline, 91 had active arthritis (median active joints=4). In this subset of children with persistent arthritis, there were no differences in age at onset, time to diagnosis or disease duration, but differences in medication use at baseline enrollment were noted (increased current IL-6 inhibitor, corticosteroid, and NSAID use and increased past use of all biologics).

## Conclusion

This study describes characteristics and medication usage of the largest sJIA cohort reported to date. Significant changes occurred in sJIA medication usage from 2010-2013, but corticosteroids are still frequently used (29.2% at enrollment). AA children have more severe disease, as do children diagnosed at a younger age. A significant proportion of children have persistent arthritis despite the use of new treatments. Further study is needed to identify predictors of persistent arthritis in order to improve treatment and outcomes in this subgroup of patients.

## Disclosure of interest

G. Janow: None declared., L. Schanberg Grant / Research Support from: Novartis, Consultant for: UCB, Lilly, S. Setoguchi Grant / Research Support from: Novartis, E. D. Mellins* Grant / Research Support from: Novartis, Five Prime, Consultant for: Ascendant, R. Schneider* Grant / Research Support from: Novartis, Consultant for: Novartis, Roche, Y. Kimura* Grant / Research Support from: Novartis, Consultant for: Novartis.

